# Relationships Between Metabolic Body Composition Status and Rapid Kidney Function Decline in a Community-Based Population: A Prospective Observational Study

**DOI:** 10.3389/fpubh.2022.895787

**Published:** 2022-06-03

**Authors:** Shao-Chi Chu, Po-Hsi Wang, Kuan-Ying Lu, Chia-Chun Ko, Yun-Hsuan She, Chin-Chan Lee, I-Wen Wu, Chiao-Yin Sun, Heng-Jung Hsu, Heng-Chih Pan

**Affiliations:** ^1^Division of Nephrology, Department of Internal Medicine, Keelung Chang Gung Memorial Hospital, Keelung, Taiwan; ^2^College of Medicine, Chang Gung University, Taoyuan, Taiwan; ^3^Community Medicine Research Center, Keelung Chang Gung Memorial Hospital, Keelung, Taiwan; ^4^Graduate Institute of Clinical Medicine, College of Medicine, National Taiwan University, Taipei, Taiwan

**Keywords:** metabolic body composition status, metabolic syndrome, obesity, rapid kidney function decline, chronic kidney disease

## Abstract

Obesity and metabolic syndrome are strong risk factors for incident chronic kidney disease (CKD). However, the predictive accuracy of metabolic body composition status (MBCS), which combines the status of obesity and metabolic syndrome, for rapid kidney function decline (RKFD) is unclear. The aim of this study was to investigate the relationship between MBCS and RKFD in a healthy population in a prospective community-based cohort study. In the current study, we followed changes in renal function in 731 people residing in northern Taiwan for 5 years. The participants were divided into four groups according to their MBCS, including metabolically healthy normal weight (MHNW), metabolically healthy overweight (MHOW), metabolically unhealthy normal weight (MUNW), and metabolically unhealthy overweight (MUOW). We evaluated traditional risk factors for CKD and metabolic profiles. The primary outcome was RKFD, which was defined as a 15% decline in estimated glomerular filtration rate (eGFR) within the first 4 years, and a reduction in eGFR which did not improve in the 5th year. During the study period, a total of 731 participants were enrolled. The incidence of RKFD was 17.1% (125/731). Multiple Cox logistic regression hazard analysis revealed that age, cerebrovascular accident, eGFR, urine albumin-to-creatinine ratio, use of painkillers, depressive mood, MUNW and MUOW were independent predictors of RKFD. After adjusting for age, sex, eGFR and total cholesterol, the participants with MUNW and MUOW had higher hazard ratios (HRs) for RKFD [HR: 2.19, 95% confidence interval (CI): 1.22–3.95 for MUNW; HR: 1.86, 95% CI: 1.21–2.87 for MUOW] than those with MHNW. Similar results were also observed in subgroup analysis of those aged above 65 years. On the basis of the results of this study, we conclude that MBCS was independently associated with RKFD, especially in the older adults. On the basis of our results, we suggest that MUNW and MUOW should be considered as risk factors for RKFD.

## Introduction

Chronic kidney disease (CKD) is a major global health issue, with a global prevalence of ~13% (1). The incidence of CKD is increasing along with the aging population. Many risk factors have been associated with CKD, including hypertension, diabetes mellitus, hyperlipidemia, smoking and obesity (2). In clinical practice, the progression of renal impairment is highly variable, and a rapid decline in kidney function in patients with type 2 diabetes can lead to advanced diabetic kidney disease within months. Various risk factors for rapid kidney function decline (RKFD) in diabetic patients have been reported, including ethnic and genetic factors, lifestyle and health behaviors, poor sugar and lipid control, dyslipidemia, macroalbuminuria, and biochemical abnormalities (3). However, the risk factors for RKFD in healthy people are still largely unknown.

Both obesity and metabolic syndrome have been shown to be strong independent risk factors for the development of cardiovascular disease and CKD (4, 5). Although body mass index (BMI) is the most common measure of overall obesity, not all obese people have metabolic dysfunction (6). The distribution of body fat has been identified as an important factor in people with metabolic dysfunction, and visceral adipose tissue has been more closely associated with complications (7, 8). Many methods used to evaluate body fat distribution, such as computed tomography (CT) and magnetic resonance imaging (MRI), are expensive and not feasible in clinical practice. The concept of metabolic body composition status (MBCS), which may reflect different patterns of body fat distribution (9–11), has been proposed to describe six different phenotypes (12), namely metabolically healthy normal weight (MHNW), metabolically healthy overweight (MHOW), metabolically healthy obesity (MHO), metabolically unhealthy normal weight (MUNW), metabolically unhealthy overweight (MUOW), and metabolically unhealthy obesity (MUO). In recent years, several studies have indicated the importance of MBCS in assessing the risk of diabetes and cardiovascular disease (13, 14). Emerging evidence has also described a relationship between MBCS and incident CKD (15–18), however the results of previous studies have been inconsistent.

To the best of our knowledge, no prospective clinical study has investigated the relationship between MBCS and RKFD in healthy individuals. To address this issue, the purpose of this study was to examine whether different phenotypes of MBCS have different prognostic information for the risk of RKFD in a community-based cohort study in Taiwan.

## Materials and Methods

### Ethics Statement

This study was conducted in compliance with the ethical principles mentioned in the Declaration of Helsinki and was consistent with Good Clinical Practice guidelines and local regulatory requirements. The participants were invited to join this study on the day of health screening. Trained nurses evaluated the mental status of all participants during the screening, and then proceeded with informed consent procedures. Written informed consent was obtained from all participants before entering the study. This study was approved by the Institutional Review Board of Chang Gung Memorial Hospital (Approval No. 201800277B0C601).

### Patient Information and Data Collection

From January 2013 to December 2018, this longitudinal, community-based cohort study was conducted in four districts of northeastern Taiwan, namely Wanli, Anle, Ruifang, and Gongliao. The participants were recruited from a community outreach health screening program, which included a physical examination, blood and urine laboratory tests, along with a questionnaire survey. The standardized questionnaire was conducted by a trained team of interviewers, and asked about drinking, smoking, and betel nut chewing habits, exercise, medication history (oral hypoglycemic agents, insulin injections, statins, herbs and hormones), family history, and physical and mental health status (short form health survey, sleep quality survey, depression survey, and health knowledge). All of the participants signed informed consent forms.

Blood and urine samples were collected from each participant, and basic physical measurements including blood pressure (mmHg), body weight (kg) and body height (cm) were performed. The laboratory data included biochemistry markers, inflammatory markers, and metabolic syndrome-associated markers. Albuminuria and proteinuria were assessed using the urine samples according to urine albumin creatinine ratio (UACR) and urine protein creatinine ratio (UPCR). Participants with systemic disorders, such as hypertension, hyperlipidemia, and CKD, were recorded. The exclusion criteria were: participants aged ≤ 30 years, participants who were lost to follow-up, participants or their next of kin who declined to be enrolled in the study, and those who had undergone organ transplantation or renal replacement therapy before entering this study. Follow-up examinations were conducted annually, during which the physical measurements, blood and urine laboratory test data, and answers to the survey, were recorded again.

### Follow-Up

During the recruitment period, 6,734 individuals completed the baseline survey and were invited to attend yearly follow-up visits. Of those invited, 752 attended and 5,982 declined. At the end of the study, 731 patients had successfully completed 5 years of annual follow-up, 14 had died, and 7 had been lost to follow-up.

### CKD

According to the National Kidney Foundation K/DOQI classification for CKD, CKD was defined as persistent proteinuria or a decreased eGFR of <60 mL/min/1.73 m^2^, determined using the abbreviated Modification of Diet in Renal Disease equation (19).

### Metabolic Syndrome

According to the National Cholesterol Education Program (NCEP) Adult Treatment Panel III (ATP III) Guidelines, metabolic syndrome is defined as the presence of three out of five of the following criteria:

- Waist circumference of ≥90 cm in men and ≥80 cm in women in the modified Asian criteria
- Triglyceride level ≥ 150 mg/dL or treatment for elevated triglycerides
- High-density lipoprotein (HDL) cholesterol level < 40 mg/dL in men or <50 mg/dL in females, or treatment for low HDL cholesterol
- Blood pressure ≥ 130/85 mmHg or treatment for hypertension
- Fasting glucose ≥ 100 mg/dL or previously diagnosed type 2 diabetes

### Homeostasis Model Assessment-Insulin Resistance

The HOMA-IR was used to quantify insulin resistance and was calculated as: fasting insulin (μIU/ mL) × fasting glucose (mg/dL)/405. An increased HOMA-IR score denotes decreased insulin sensitivity (20, 21).

### Body Mass Index

BMI was calculated as the body weight divided by the square of the height (kg/m^2^).

### Metabolic Body Composition Status

The participants were divided into four groups according to the metabolic body composition status (Supplementary Table 1):

- Metabolically healthy normal weight (MHNW): HOMA-IR score < 2.5 without metabolic syndrome, BMI < 24 kg/m^2^
- Metabolically healthy overweight (MHOW): HOMA-IR score < 2.5 without metabolic syndrome, BMI ≥ 24 kg/m^2^
- Metabolically unhealthy normal weight (MUNW): HOMA-IR score ≥ 2.5 or with metabolic syndrome, BMI < 24 kg/m^2^
- Metabolically unhealthy overweight (MUOW): HOMA-IR score ≥ 2.5 or with metabolic syndrome, BMI ≥ 24 kg/m^2^

Different from the WHO recommendations, we used the cut-off standard recommended by the Ministry of Health and Welfare in Taiwan, and defined BMI ≥ 24 kg/m^2^ as overweight (22, 23).

### 25(OH)D

Serum level of 25(OH)D was measured using an electro-chemiluminescence immunoassay (Cobas^®^ Vitamin D3 assay, Roche Diagnostics GmbH, Mannheim, Germany) with an interassay coefficient of variation of 2.2–13.6% (19).

### Adiponectin and Leptin

Assays for Biomarkers An enzyme-linked immunosorbent assay (ELISA) was used to quantify the concentration of serum biomarkers. The serum concentrations of leptin and adiponectin were assayed using commercial ELISA kits from Boster (Pleasanton, CA, United States) (24).

### High-Sensitivity C-Reactive Protein

The HS-CRP level was assayed using a commercial ELISA kit from Roche (Basel, Switzerland), according to the manufacturers' instructions (24).

### Tumor Necrosis Factor Alpha

A quantitative sandwich enzyme immunoassay technique was used for the TNF-α assay according to the manufacturer's instructions (Immunite 1000 LKNF1, Siemens Medical Solutions Diagnostics, Llanberis, UK) (25).

### Outcome Assessment

Beginning from the index date, all eligible participants were followed up for 5 years. The primary outcome of this study was RKFD. In the literature, RKFD was defined as a decline ≥ 30% within 10 years or an absolute annual loss ≥ 3 mL/min/1.73 m^2^ (26). In this 5-year longitudinal follow-up study, RKFD was defined as a 15% decline in estimated glomerular filtration rate (eGFR) within the first 4 years, and a reduction in eGFR which did not improve in the 5th year.

### Statistical Analysis

Continuous variables were summarized using means and standard deviations (SDs). All variables were tested for normal distribution using the Kolmogorov-Smirnov test. The Student's *t*-test was used to compare the means of continuous variables and normally distributed data; otherwise, the Mann-Whitney U-test was used. The study participants were divided into four groups by their MBCS. ANOVA was also employed to compare the means of continuous variables and normally distributed data; otherwise, the Kruskal-Wallis test was used. Categorical data were tested using the chi-square test. Risk factors were assessed with univariable Cox regression analysis. In addition, variables with statistical significance (*p* < 0.05) in the univariable analysis were included in multivariable analysis by applying multiple logistic regression based on forward elimination of data. Cumulative survival curves as a function of time were produced using the Kaplan-Meier method, and compared using the log rank test. All statistical tests were two-tailed, and a value of *p* < 0.05 was considered to be statistically significant. Data were analyzed using SPSS version 22.0 for Windows 10 (SPSS, Inc., Chicago, IL, USA).

## Results

### Baseline Characteristics of the Study Population

A total of 731 participants were included in this study (mean age 57.2 years; 29.1% male). The baseline characteristics of the participants with and without RKFD are shown in Table 1. The incidence of RKFD was 17.1% (125/731), the prevalence of CKD was 2.1%, and diabetes mellitus was present in 9.1% of the study population. There were no significant differences in age and the prevalence of CKD between those with and without RKFD. The RKFD group had a higher percentage of females and higher prevalence rates of diabetes mellitus and cerebrovascular accident (CVA). With regards to the use of medications, the RKFD group was more likely to regularly use oral hypoglycemic agents (OHAs) and painkillers. The RKFD group also had significantly higher levels of eGFR and UACR. With regards to the factors associated with metabolic syndrome, there were no significant differences in BMI, blood pressure, fasting glucose, triglycerides, HDL cholesterol, leptin, adiponectin, tumor necrosis factor alpha (TNF-α) and vitamin D between the participants with and without RKFD. The RKFD group had significantly higher glycated hemoglobin (HbA1C), insulin, and HOMA-IR, and higher percentages of MUNW and MUOW; while the non-RKFD group had significantly higher total cholesterol and low-density lipoprotein (LDL) cholesterol, and higher percentage of MHNW. We also compared the social psychology variables of the study population (Supplementary Table 2). The RKFD group tended to have a lower level of education and less alcohol consumption, and higher percentages of vegetarians and depressive mood.

**Table 1:** Baseline characteristics of the study population.

	**Total** **(*n* = 731)**	**RKFD** **(*n* = 125)**	**No RKFD** **(*n* = 606)**	***p*-value**
**Demographics**				
Age, years	57.2 ± 10.3	58.8 ± 11.3	56.8 ± 10.1	NS (0.070)
Male gender, *n* (%)	213 (29.1%)	26 (20.8%)	187 (30.9%)	**0.024**
Hypertension, *n* (%)	176 (24.1%)	37 (29.6%)	139 (23.0%)	NS (0.135)
DM, *n* (%)	66 (9.1%)	19 (15.2%)	47 (7.8%)	**0.015**
CKD, *n* (%)	15 (2.1%)	4 (3.2%)	11 (1.8%)	NS (0.303)
Cardiovascular disease, *n* (%)	52 (7.1%)	11 (8.8%)	41 (6.8%)	NS (0.445)
CVA, *n* (%)	5 (0.7%)	3 (2.4%)	2 (0.3%)	**0.038**
HBV, *n* (%)	81 (11.1%)	15 (12.0%)	66 (10.9%)	NS (0.755)
HCV, *n* (%)	11 (1.5%)	2 (1.6%)	9 (1.5%)	NS (1.000)
Gout, *n* (%)	30 (4.1%)	5 (4.0%)	25 (4.1%)	NS (1.000)
Autoimmune disease, *n* (%)	11 (1.5%)	2 (1.6%)	9 (1.5%)	NS (1.000)
**Biochemical and physiological profiles**				
SBP, mmHg	130.3 ± 17.8	131.1 ± 17.1	130.1 ± 17.9	NS (0.547)
BMI, kg/m^2^	24.4 ± 3.5	24.5 ± 3.4	24.3 ± 3.5	NS (0.742)
Waist circumference, cm	80.1 ± 9.3	79.7 ± 9.2	81.5 ± 9.4	**0.048**
Hgb, g/dL	13.8 ± 1.3	13.6 ± 1.4	13.8 ± 1.3	NS (0.051)
Total cholesterol, mg/dL	210.4 ± 37.2	202.6 ± 35.5	212.0 ± 37.4	**0.011**
LDL cholesterol, mg/dL	126.5 ± 32.0	120.5 ± 29.9	127.7 ± 32.3	**0.021**
HDL cholesterol, mg/dL	58.6 ± 14.8	56.8 ± 14.3	59.0 ± 14.9	NS (0.138)
Triglycerides, mg/dL	113.6 ± 73.1	114.5 ± 77.8	113.4 ± 72.2	NS (0.885)
BUN, mg/dL	12.8 ± 3.7	12.7 ± 4.0	12.8 ± 3.6	NS (0.849)
Creatinine, mg/dL	0.71 ± 0.17	0.65 ± 0.17	0.73 ± 0.17	**<0.001**
eGFR, ml/min/1.73 m^2^	97.6 ± 21.8	105.9 ± 25.3	95.9 ± 20.6	**<0.001**
Uric acid, mg/dL	5.4 ± 1.3	5.3 ± 1.3	5.4 ± 1.3	NS (0.241)
Albumin, g/dL	4.7 ± 0.3	4.7 ± 0.3	4.7 ± 0.3	NS (0.131)
GPT, U/L	25.1 ± 23.4	26.9 ± 20.5	24.7 ± 23.9	NS (0.354)
UACR, mg/g	6.9 ± 5.0	8.4 ± 5.7	6.6 ± 4.7	**0.001**
Fasting glucose, mg/dL	101.8 ± 24.3	106.5 ± 31.3	100.8 ± 22.5	NS (0.057)
HbA1C, %	5.8 ± 0.7	6.0 ± 1.1	5.7 ± 0.6	**0.017**
Insulin, μIU/ml	7.1 ± 5.5	8.4 ± 7.9	6.8 ± 4.9	**0.030**
Adiponectin, ng/ml	7.5 ± 5.4	7.9 ± 6.5	7.5± 5.1	NS (0.542)
Leptin, ng/mL	13.9 ± 9.1	14.2 ± 8.7	13.9 ± 9.1	NS (0.720)
HOMA-IR	1.9 ± 1.8	2.3 ± 2.8	1.8 ± 1.6	**0.028**
TNF-α, ng/dL	7.6 ± 5.0	7.6 ± 2.8	7.6 ± 5.4	NS (0.903)
25(OH)D, ng/mL	28.2 ± 10.2	27.1 ± 10.8	28.4 ± 10.0	NS (0.274)
HS-CRP, mg/L	0.88 (0, 30)	0.90 (0, 30)	0.88 (0, 22)	NS (0.904)
**Medications**				
OHAs, *n* (%)	60 (8.3%)	19 (15.6%)	41 (6.8%)	**0.003**
Anti-hypertensives, *n* (%)	155 (21.6%)	33 (27.0%)	122 (20.5%)	NS (0.117)
Painkillers, *n* (%)	77 (10.9%)	20 (17.1%)	57 (9.7%)	**0.023**
**Metabolic body composition status**				**0.001**
MHNW, *n* (%)	322 (44.0%)	41 (32.8%)	281 (46.4%)	
MHOW, *n* (%)	173 (23.7%)	24 (19.2%)	149 (24.6%)	
MUNW, *n* (%)	56 (7.7%)	16 (12.8%)	40 (6.6%)	
MUOW, *n* (%)	180 (24.6%)	44 (35.2%)	136 (22.4%)	

*BMI, body mass index; BUN, blood urea nitrogen; CKD, chronic kidney disease; CVA, cerebrovascular accident; DM, diabetes mellitus; eGFR, estimated glomerular filtration rate; GPT, glutamic pyruvic transaminase; Hgb, hemoglobin; HbA1C, glycated hemoglobin; HBV, hepatitis B virus; HCV, hepatitis C virus; HOMA-IR, homeostatic model assessment-insulin resistance; HDL, high-density lipoprotein; HS-CRP, high-sensitivity C-reactive protein; LDL, low-density lipoprotein; MHNW, metabolically healthy normal-weight; MHOW, metabolically healthy overweight; MUNW, metabolically unhealthy normal-weight; MUOW, metabolically unhealthy overweight; OHA, oral hypoglycemic agent; RKFD, rapid kidney function decline; SBP, systolic blood pressure; TNF-α, tumor necrosis factor alpha; UACR, urine albumin-to-creatinine ratio. Numbers in bold indicate significant difference (P < 0.05)*.

The baseline characteristics and social psychology variables of the study population according to their MBCS are presented in Tables 2, 3, respectively. The MHOW group had the highest percentage of males. The participants in the MUNW group were the oldest, but the MUOW group had the highest prevalence rates of diabetes mellitus, hypertension, cardiovascular disease and gout. There was no significant difference in the prevalence of CKD between the different MBCS groups. The MUOW group had worse metabolic profile, including the highest blood pressure, BMI, waist circumstance, triglycerides, uric acid, glutamic pyruvic transaminase (GPT), UACR, fasting glucose, HbA1C, insulin, leptin and HOMA-IR, and the lowest HDL cholesterol and adiponectin. In addition, the MUOW also had the highest inflammatory markers, including TNF-α and HS-CRP, as well as the highest use of OHAs and anti-hypertensives (Table 2). The MUNW group had the highest blood urea nitrogen level. With respect to social psychology, the MUNW and MUOW groups had significantly lower levels of education, while the MUNW group had a higher prevalence of smoking than the other groups (Table 3).

**Table 2:** Baseline characteristics of the population, stratified by metabolic body composition status.

	**MHNW** **(*n* = 322)**	**MHOW** **(*n* = 173)**	**MUNW** **(*n* = 56)**	**MUOW** **(*n* = 180)**	***P*-value**
**Demographics**					
Age, years	55.7 ± 10.2^b, c^	57.2 ± 10.3	61.3 ± 10.0^b^	58.4 ± 10.2^c^	**<0.001**
Male gender, *n* (%)	74 (23.0%)	62 (35.8%)	17 (30.4%)	60 (33.3%)	**0.010**
Hypertension, *n* (%)	41 (12.8%)	43 (24.9%)	19 (33.9%)	73 (40.8%)	**<0.001**
DM, *n* (%)	10 (3.1%)	9 (5.2%)	10 (17.9%)	37 (20.7%)	**<0.001**
CKD, *n* (%)	9 (2.8%)	3 (1.7%)	1 (1.8%)	2 (1.1%)	NS (0.618)
Cardiovascular disease, *n* (%)	14 (4.4%)	17 (9.8%)	1 (1.8%)	20 (11.2%)	**0.006**
CVA, *n* (%)	2 (0.6%)	1 (0.6%)	1 (1.8%)	1 (0.6%)	NS (0.781)
HBV, *n* (%)	34 (10.6%)	29 (16.8%)	5 (8.9%)	13 (7.3%)	**0.035**
HCV, *n* (%)	7 (2.2%)	1 (0.6%)	1 (1.8%)	2 (1.1%)	NS (0.533)
Gout, *n* (%)	7 (2.2%)	7 (4.0%)	5 (8.9%)	11 (6.1%)	**0.042**
Autoimmune disease, *n* (%)	4 (1.2%)	3 (1.7%)	1 (1.8%)	3 (1.7%)	NS (0.965)
**Biochemical and physiological profiles**					
SBP, mmHg	123.9 ± 16.6^b, c^	128.1 ± 15.2	139.5 ± 15.2^b^	140.8 ±16.9^c^	**<0.001**
BMI, kg/m^2^	21.6 ± 1.7 ^a, b, c^	26.5 ± 2.2^a, d, e^	22.5 ± 1.3^b, d, f^	27.9 ± 2.7^c, e, f^	**<0.001**
Waist circumference, cm	73.6 ± 6.5^a, b, c^	83.5 ± 6.8^a, d, e^	79.3 ± 6.4^b, d, f^	88.4 ± 7.6^c, e, f^	**<0.001**
Hgb, g/dL	13.6 ± 1.3	13.9 ± 1.3	13.8 ± 1.3	13.9 ± 1.4	NS (0.084)
Total cholesterol, mg/dL	212.5 ± 33.0	208.9 ± 38.2	216.3 ± 47.4	206.1 ± 39.6	NS (0.211)
LDL cholesterol, mg/dL	124.6 ± 28.9	128.7 ± 33.2	133.2 ± 38.9	125.8 ± 33.7	NS (0.283)
HDL cholesterol, mg/dL	65.5 ± 15.3^a, b, c^	57.5 ± 10.4^a^	52.2 ± 16.0^b^	49.2 ± 10.3^c^	**<0.001**
Triglycerides, mg/dL	86.9 ± 46.4^b, c^	101.0 ± 51.5^d, e^	142.9 ± 78.3^b, d^	164.4 ± 95.9^c, e^	**<0.001**
BUN, mg/dL	12.3 ± 3.5^a, b^	13.3 ± 3.8^a^	13.8 ± 3.9^b^	12.9 ± 3.8	**0.002**
Creatinine, mg/dL	0.7 ± 0.2	0.7 ± 0.2	0.7 ± 0.2	0.7 ± 0.2	NS (0.078)
eGFR, ml/min/1.73 m^2^	98.9 ± 21.2	97.0 ± 21.4	93.5 ± 23.8	97.3 ± 22.4	NS (0.361)
Uric acid, mg/dL	4.9 ± 1.1^a, b, c^	5.6 ± 1.2 ^a^	5.7 ± 1.7 ^b^	5.9 ± 1.3^c^	**<0.001**
Albumin, g/dL	4.7 ± 0.3	4.7 ± 0.3	4.7 ± 0.3	4.7 ± 0.3	NS (0.078)
GPT, U/L	20.5 ± 11.1^a, c^	27.4 ± 34.5^a^	25.0 ± 13.7	31.1 ± 26.9^c^	**<0.001**
UACR, mg/g	6.5 ± 4.8^c^	6.0 ± 4.8^e^	7.9 ± 5.3	8.1 ± 5.0^c, e^	**<0.001**
Fasting glucose, mg/dL	94.1 ± 10.4	96.2 ± 9.7	114.8 ± 26.8	116.9 ± 38.6	**<0.001**
HbA1C, %	5.5 ± 0.4^b, c^	5.6 ± 0.4^d, e^	6.1 ± 0.8^b, d^	6.2 ± 1.0^c, e^	**<0.001**
Insulin, μIU/ml	4.5 ± 1.8^b, c^	6.0 ± 2.1^d, e^	10.4 ± 7.2^b, d^	11.7 ± 7.9^c, e^	**<0.001**
Adiponectin, ng/ml	8.9 ± 5.7 ^a, b, c^	7.2 ± 4.4^a^	6.1 ± 5.1^b^	5.9 ± 5.0^c^	**<0.001**
Leptin, ng/mL	11.0 ± 7.4^a, c^	15.1 ± 8.2^a, e^	12.8 ± 7.9^f^	18.3 ± 10.9^c, e, f^	**<0.001**
HOMA-IR	1.1 ± 0.4^b, c^	1.4 ± 0.5^d, e^	2.9 ± 2.3^b, d^	3.3 ± 2.7^c, e^	**<0.001**
TNF-α, ng/dL	7.0 ± 2.1	9.3 ± 0.8	2.4 ± 0.4	2.6 ± 0.2	NS (0.098)
25(OH)D, ng/mL	27.5 ± 10.2	30.0 ± 10.5	29.6 ± 10.0	27.3 ± 9.8	NS (0.111)
HS-CRP, mg/L	1.3 ± 2.9^c^	1.8 ± 2.6	2.7 ± 4.2	2.1 ± 2.3^c^	**<0.001**
**Medications**					
OHA, *n* (%)	6 (1.9%)	9 (5.2%)	10 (17.9%)	35 (19.9%)	**<0.001**
Anti-hypertensives, *n* (%)	34 (10.8%)	36 (21.2%)	18 (32.1%)	67 (38.1%)	**<0.001**
Painkillers, *n* (%)	33 (10.6%)	23 (13.5%)	6 (10.7%)	15 (8.9%)	NS (0.590)

*BMI, body mass index; BUN, blood urea nitrogen; CKD, chronic kidney disease; CVA, cerebrovascular accident; DM, diabetes mellitus; eGFR, estimated glomerular filtration rate; GPT, glutamic pyruvic transaminase; Hgb, hemoglobin; HbA1C, glycated hemoglobin; HBV, hepatitis B virus; HCV, hepatitis C virus; HOMA-IR, homeostatic model assessment-insulin resistance; HDL, high-density lipoprotein; HS-CRP, high-sensitivity C-reactive protein; LDL, low-density lipoprotein; MHNW, metabolically healthy normal-weight; MHOW, metabolically healthy overweight; MUNW, metabolically unhealthy normal-weight; MUOW, metabolically unhealthy overweight; OHA, oral hypoglycemic agent; SBP, systolic blood pressure; TNF-α, tumor necrosis factor alpha; UACR, urine albumin-to-creatinine ratio*.

*The mean values of each group with the same superscript letter (a–e) are significantly different according to one-way ANOVA with post-hoc Bonferroni test. In all cases the difference is significant with p <0.05.

**Table 3:** Social psychology variables of the study population stratified by metabolic body composition status.

	**MHNW (*n* = 322)**	**MHOW** **(*n* = 173)**	**MUNW** **(*n* = 56)**	**MUOW** **(*n* = 180)**	***p*-value**
Education level					**0.010**
None, *n* (%)	11 (3.4%)	9 (5.2%)	4 (7.1%)	7 (3.9%)	
Elementary school, *n* (%)	52 (16.1%)	48 (27.7%)	16 (28.6%)	56 (31.1%)	
Junior high school, *n* (%)	52 (16.1%)	32 (18.5%)	9 (16.1%)	25 (13.9%)	
Senior high school, *n* (%)	127 (39.4%)	46 (26.6%)	17 (30.4%)	56 (31.1%)	
College or university, *n* (%)	70 (21.7%)	35 (20.2%)	7 (12.5%)	26 (14.4%)	
Graduate school, *n* (%)	7 (2.2%)	3 (1.7%)	3 (5.4%)	6 (3.3%)	
**Substance habits**					
Smoking, *n* (%)	44 (13.8%)	39 (22.5%)	14 (25.0%)	33(18.8%)	0.042
Betel nut, *n* (%)	10 (3.1%)	7 (2.3%)	1 (1.8%)	9 (5.0%)	NS (0.617)
Alcohol, *n* (%)	70 (21.7%)	53 (30.6%)	11 (19.6%)	44(24.4%)	NS (0.183)
**Dietary habits**					NS (0.372)
Meat diet, *n* (%)	284 (88.2%)	153 (88.4%)	45 (80.4%)	159 (88.3%)	
Vegetarian food, *n* (%)	35 (10.9%)	19 (11.0%)	10 (17.9%)	17 (9.4%)	
Depressive mood, *n* (%)	39 (12.1%)	22 (12.7%)	5 (8.9%)	29 (16.1)	NS (0.458)

*MHNW, metabolically healthy normal-weight; MHOW, metabolically healthy overweight; MUNW, metabolically unhealthy normal-weight; MUOW, metabolically unhealthy overweight. Numbers in bold indicate significant difference (P < 0.05)*.

**Figure 1:**
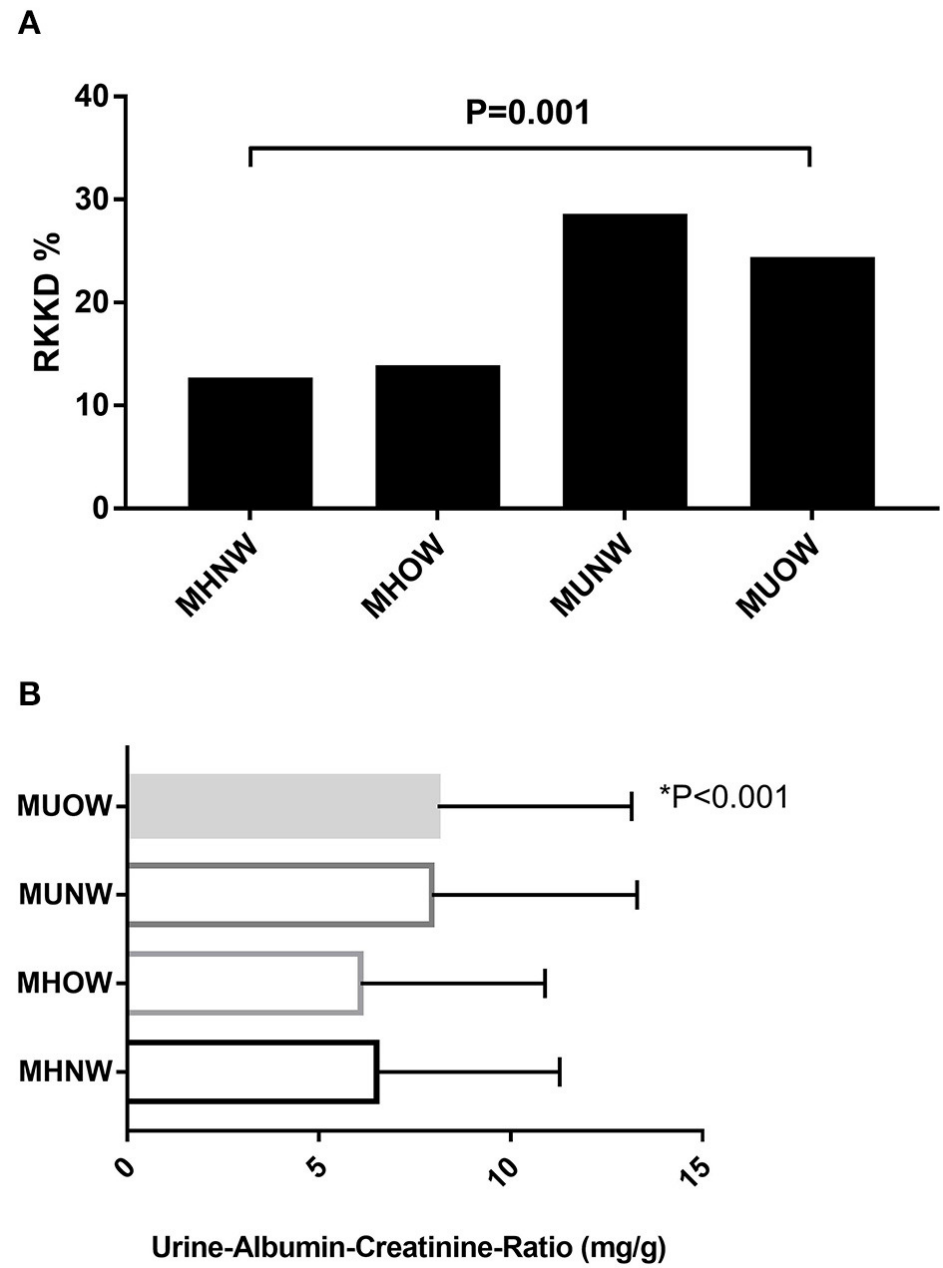
**(A)** Overall RKFD rate according to MBCS. **(B)** Overall UACR according to MBCS. MBCS, metabolic body composition status; RKFD, rapid kidney function decline; UACR, urinary albumin-creatinine ratio.

The RKFD rate and UACR according to MBCS are depicted in Figures 1A,B, respectively. The MUOW group had the highest UACR, while the MUNW group had the highest RKFD rate.

### Risk Factors for RKFD

The univariable Cox proportional hazards analysis identified that 16 (Table 4) of the 44 variables (Tables 1, 3) were good prognostic indicators. In the Cox regression multivariable analysis, age, CVA, eGFR, UACR, use of painkillers, depressive mood, MUNW and MUOW were significant independent prognosticators for RKFD. CVA had the highest hazard ratio (HR) for RKFD [HR: 3.48, 95% confidence interval (CI): 1.08–11.17], following by MUNW (HR: 2.24, 95% CI: 1.26–3.99), depression (HR: 1.95, 95% CI: 1.23–3.09), use of painkillers (HR: 1.72, 95% CI: 1.04–2.85), and MUOW (HR: 1.61, 95% CI: 1.06–2.45). UACR, eGFR and age were also reliable prognostic indicators.

**Table 4:** Univariate Cox regression analysis for the risk of RKFD according to baseline characteristics.

**Parameter**	**HR (95% CI)**	***P*-value**
**Univariable hazard analysis**		
Age, years	1.02 (1.00–1.04)	0.053
Male gender, *n*	0.62 (0.40–0.95)	**0.029**
DM, *n*	1.91 (1.17–3.11)	**0.010**
Hypertension, *n*	1.36 (0.93–2.00)	0.116
CKD, *n*	1.78 (0.66–4.82)	0.256
Cardiovascular disease, *n*	1.26 (0.68–2.33)	0.471
CVA, *n*	4.24 (1.35–13.32)	**0.014**
Creatinine, mg/dL	0.07 (0.02–0.23)	**<0.001**
eGFR, ml/min/1.73 m^2^	1.02 (1.01–1.02)	**<0.001**
Hgb, g/dL	0.88 (0.78–1.00)	0.053
Total cholesterol, mg/dL	0.99 (0.99–1.00)	**0.014**
LDL, mg/dL	0.99 (0.99–1.00)	**0.027**
UACR, mg/g	1.06 (1.03–1.09)	**<0.001**
HbA1C, %	1.42 (1.18–1.70)	**<0.001**
Insulin, μIU/ml	1.03 (1.01–1.05)	**0.005**
Painkillers, *n*	1.72 (1.06–2.79)	**0.027**
Education level, *n*	0.79 (0.68–0.91)	**0.001**
Depressive mood, *n*	1.87 (1.22–2.87)	**0.004**
TNF-α, log	1.54 (0.41–5.80)	0.523
HS-CRP, log	1.01 (0.66–1.55)	0.977
MHNW, *n*	0.60 (0.42–0.88)	**0.008**
MHOW, *n*	0.75 (0.48–1.17)	0.198
MUNW, *n*	1.84 (1.09–3.11)	**0.023**
MUOW, *n*	1.75 (1.21–2.52)	**0.003**
**Multivariable hazard analysis**		
Age, years	1.02 (1.003–1.04)	**0.027**
CVA, *n*	3.48 (1.08–11.17)	**0.036**
eGFR, ml/min/1.73 m^2^	1.02 (1.01–1.03)	**<0.001**
Total cholesterol, mg/dL	0.995 (0.99–1.00)	0.051
UACR, mg/g	1.05 (1.01–1.08)	**0.007**
Painkillers, *n*	1.72 (1.04–2.85)	**0.035**
Depressive mood, *n*	1.95 (1.23–3.09)	**0.005**
MUNW, *n*	2.24 (1.26–3.99)	**0.006**
MUOW, *n*	1.61 (1.06–2.45)	**0.026**

*CKD, chronic kidney disease; CVA, cerebrovascular accident; DM, diabetes mellitus; eGFR, estimated glomerular filtration rate; Hgb, hemoglobin; HbA1C, glycated hemoglobin; HS-CRP, high-sensitivity C-reactive protein; LDL, low-density lipoprotein; MHNW, metabolically healthy normal-weight; MHOW, metabolically healthy overweight; MUNW, metabolically unhealthy normal-weight; MUOW, metabolically unhealthy overweight; RKFD, rapid kidney function decline; TNF-α, tumor necrosis factor alpha; UACR, urine albumin-to-creatinine ratio. Numbers in bold indicate significant difference (P < 0.05)*.

### Association Between MBCS and RKFD

**Figure 2:**
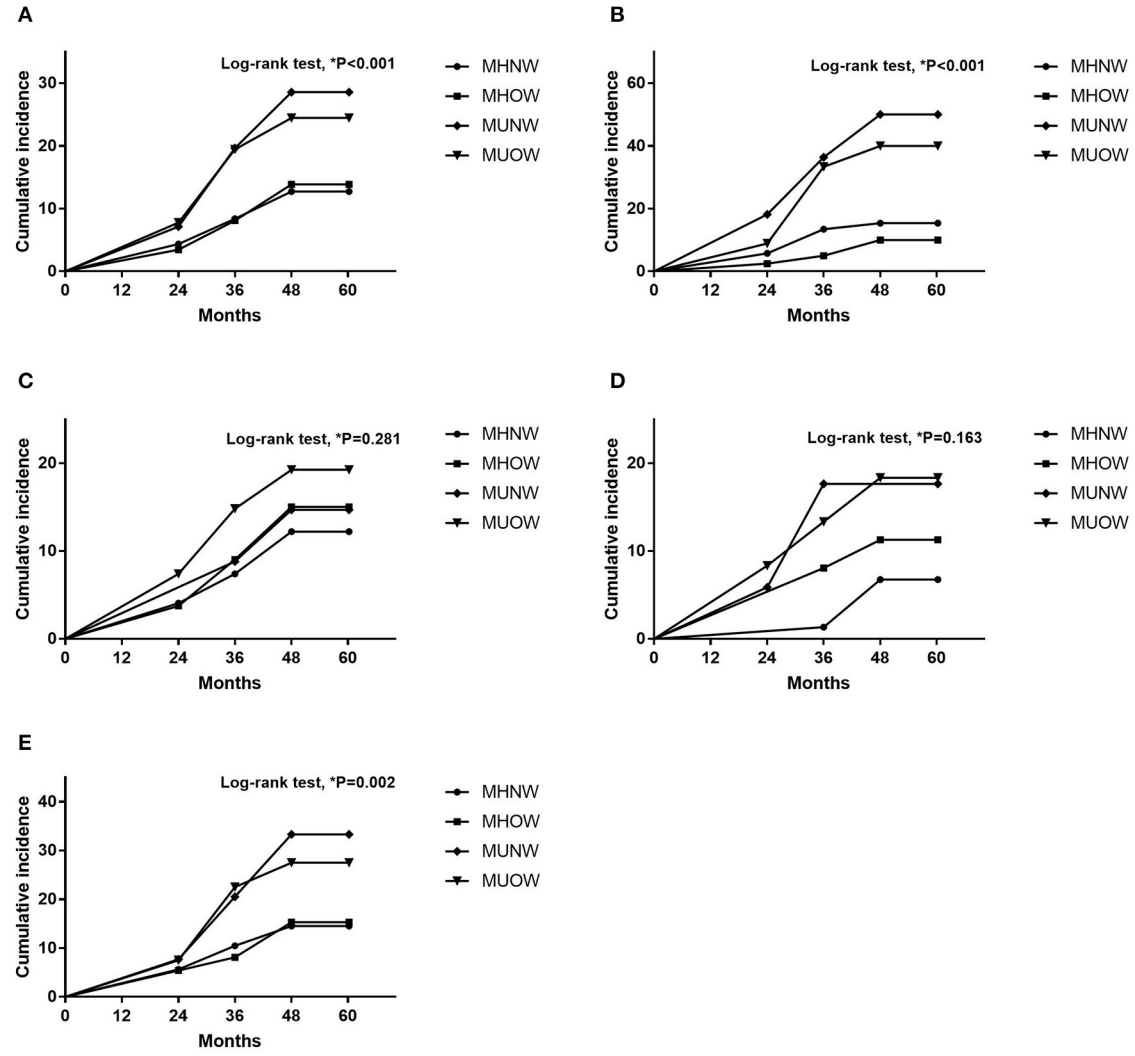
Cumulative incidence according to MBCS **(A)** Overall population. **(B)** Participants ≥ 65 years old. **(C)** Participants <65 years old. **(D)** Male participants. **(E)** Female participants. MBCS, metabolic body composition status.

Table 5 presents the associations of MBCS with RKFD in unadjusted and adjusted models. The effects of adjusting for age, sex, eGFR and total cholesterol were investigated. Compared with the MHNW group, the adjusted HRs of the MUNW and MUOW groups for RKFD were 2.19 (95% CI: 1.22–3.95, *p* = 0.009) and 1.86 (95% CI: 1.21–2.87, *p* = 0.005), respectively. In subgroup analysis according to age, similar results were observed in the adjusted model in those **≥** 65 years of age (MHNW: reference; HR: 3.15, 95% CI: 1.26–7.88 for MUNW; HR: 2.45, 95% CI: 1.05–5.74 for MUOW), while this trend was not statistically significant in those <65 years. When the population was stratified by sex, the adjusted HR of the MUOW group for RKFD was 3.03 (95% CI: 1.05–8.75, *p* = 0.041) in the males. The adjusted HRs of the MUNW and MUOW groups for RKFD were 1.96 (95% CI: 1.02–3.75, *p* = 0.043) and 1.64 (95% CI: 1.01–2.66, *p* = 0.044) in the females, respectively. Moreover, the HR in the MHOW group was statistically non-significant in both overall analysis and subgroup analysis of sex and gender. Additional analysis adjusting for education level, alcohol consumption, dietary habits, and depressive mood resulted in similar findings on the effect of MBCS on RKFD (Supplementary Table 3). Figure 2 illustrate the cumulative incidence of RKFD in the study population according to MBCS. As shown in Figure 1, the individuals with MUNW and MUOW had significantly higher incidence rates of RKFD than the individuals with MHNW in the overall cohort. This trend was also statistically significant in individuals **≥** 65 years old, and in females during the study period.

**Table 5:** Combined effects of sex, age, and MBCS on RKFD.

	**Number**	**RKFD rate**	**Unadjusted model**		**Adjusted model**	
			**HR (95% CI)**	***p*-value**	**HR (95% CI)**	***p*-value**
**Total**						
MHNW	322	41 (12.7%)	1 (reference)	1 (reference)	1 (reference)^a^	1 (reference) ^a^
MHOW	173	24 (13.9%)	1.09 (0.66–1.80)	0.752	1.10 (0.66–1.83)	0.706
MUNW	56	16 (28.6%)	**2.36 (1.33–4.21)**	**0.004**	**2.19 (1.22–3.95)**	**0.009**
MUOW	180	44 (24.4%)	**2.03 (1.32–3.10)**	**0.001**	**1.86 (1.21–2.87)**	**0.005**
**Males**						
MHNW	74	5 (6.8%)	1 (reference)	1 (reference)	1 (reference)^b^	1 (reference)^b^
MHOW	62	7 (11.3%)	1.71 (0.54–540)	0.358	1.76 (0.56–5.56)	0.335
MUNW	17	3 (17.6%)	2.85 (0.68–11.94)	0.151	3.79 (0.88–16.42)	0.075
MUOW	60	11 (18.3%)	**2.95 (1.02–8.48)**	**0.045**	**3.03 (1.05–8.75)**	**0.041**
**Females**						
MHNW	248	36 (14.5%)	1 (reference)	1 (reference)	1 (reference)^b^	1 (reference)^b^
MHOW	111	17 (21.4%)	1.05 (0.59–1.86)	0.880	1.00 (0.56–1.78)	1.000
MUNW	39	13 (33.3%)	**2.40 (1.27–4.52)**	**0.007**	**1.96 (1.02–3.75)**	**0.043**
MUOW	120	33 (27.5%)	**1.99 (1.24–3.19)**	**0.004**	**1.64 (1.01–2.66)**	**0.044**
**≥65 years old**						
MHNW	52	8 (15.4%)	1 (reference)	1 (reference)	1 (reference)^c^	1 (reference)^c^
MHOW	40	4 (10.0%)	0.63 (0.19–2.07)	0.442	0.65 (0.19–2.17)	0.484
MUNW	22	11 (50.0%)	3.73 (1.50–9.28)	**0.005**	**3.15 (1.26–7.88)**	**0.014**
MUOW	45	18 (40.0%)	2.80 (1.22–6.45)	**0.015**	**2.45 (1.05–5.74)**	**0.039**
**<65 years old** ^c^						
MHNW	270	33 (12.2%)	1 (reference)	1 (reference)	1 (reference)^c^	1 (reference)^c^
MHOW	133	20 (15.0%)	1.24 (0.71–2.16)	0.454	1.26 (0.72–2.19)	0.425
MUNW	34	5 (14.7%)	1.19 (0.47–3.05)	0.714	1.37 (0.53–3.52)	0.512
MUOW	135	26 (19.3%)	1.64 (0.98–2.75)	0.059	1.63 (0.97–2.74)	0.065

a *Adjusted for age, sex, eGFR, total cholesterol*.

b *Adjusted for age, eGFR, total cholesterol*.

c *Adjusted for sex, eGFR, total cholesterol*.

*MBCS, metabolic body composition status; RKFD, rapid kidney function decline. Numbers in bold indicate significant difference (P < 0.05)*.

## Discussion

In this prospective community-based study, we characterized the association between MBCS and RKFD. The incidence of RKFD in our study population was 17.1% (125/731), which is consistent with a previous study (27). The MUOW group had the worst metabolic profile, while the MUNW and MUOW groups had significantly lower levels of education compared to the other groups. Cox logistic regression hazard analysis revealed that age, CVA, eGFR, UACR, use of painkillers, depressive mood, MUNW and MUOW were independent predictors of RKFD. After adjusting for age, sex, eGFR, and total cholesterol, the individuals in the MUNW and MUOW groups had higher risks of RKFD (MHNW: reference; HR: 1.10, 95% CI: 0.66–1.83 for MHOW; HR: 2.19, 95% CI:1.22–3.95 for MUNW; HR: 1.86, 95% CI: 1.21–2.87 for MUOW) than those in the MHNW group, especially in the older adults.

Body fat distribution, plays an important role in multiple comorbidities, and it is quite different in different MBCS (9, 11). Visceral adipose tissue has been reported to be more associated with the complications of obesity than subcutaneous adipose tissue (7, 8). Stefan et al. reported that the occurrence of metabolically unhealthy status, regardless of MUNW or MUOW, was associated with a 3-fold higher risk of all-cause mortality and/or cardiovascular events, and that MUNW was associated with low amount of gluteofemoral fat mass (28). Gluteofemoral fat mass serves as a healthy sink to store excess fat during weight gain and prevent ectopic lipid deposition (9). Consequently, a lower amount of gluteofemoral fat mass may result in increased ectopic lipid deposition and increased lipid accumulation in the kidneys, which may then contribute to podocyte apoptosis, heavy proteinuria, and renal damage (29). Moreover, Stefan et al. also reported an association between MUOW with high visceral fat tissue and liver fat accumulation, which may further increase the risk of metabolic dysfunction and renal impairment (28). As demonstrated in the present study, both MUNW and MUOW were associated with higher UACR and markedly increased risks of RKFD than MHNW and MHOW.

Although overweight/obesity according to BMI is a known risk factor for many comorbidities, Meigs et al. reported that both metabolic and cardiovascular outcomes in patients with MUNW were significantly higher than in those with MHOW (13). This finding is consistent with our results, which showed that the MUNW group had a higher incidence of RKFD than the MHOW group (MUNW vs. MHOW: 28.6 vs. 13.9%; *p* = 0.001). Our findings are consistent with those of Meigs et al., in that overweight only increased the risk in the presence of metabolic syndrome or insulin resistance. Furthermore, aging is associated with a decrease in lean mass and an increase in fat mass, which increases body fat distribution, and hence BMI is not an accurate indicator of total body adiposity in older adults (30, 31). In the current study, we also found that metabolic unhealthy status was associated with an increased risk of RKFD in the older adults, regardless of weight status. Of note, a previous investigation also reported that the incidence of cardiovascular disease in patients with MUNW was even higher than in those with MUOW (13), which may explain why the participants with MUNW had a higher risk of RKFD than those with MUOW in our study.

We also found that depressive mood was associated with an even higher risk of RKFD than the traditional risk factors, including age, eGFR, UACR and use of painkillers (32). Comorbid depression has been reported to be a risk factor for incident CKD in diabetes patients (33) and to increase the risk of progression to ESRD or death in CKD patients (34). In the present study, we further demonstrated a strong independent association between depression and RKFD in a healthy population. Multiple mechanisms may link depression with a higher risk of RKFD, including an impaired ability to perform self-care, lack of physical exercise, a higher level of low-grade systemic inflammation, increase in sympathetic nervous system, and upregulation of the hypothalamic–pituitary–adrenal axis (33). Further well-powered research is needed to study this issue. Sex differences in the risk of CKD progression have also been well described (35, 36). Several studies have reported that for the same BMI, although women typically had about 10% more body fat than men, the women had a lower amount of visceral adipose tissue (37, 38). Compared to the central obesity in men, women often have a pear-shaped body fat distribution and more gluteofemoral adipose tissue, which may provide a safe lipid reservoir for excess fat (39). In the present study, we also demonstrated that, compared with MHNW, males with MUOW seemed to have an even higher risk of RKFD than females with MUOW (HR: 3.03, 95% CI: 1.05–8.75 for males; HR: 1.64, 95% CI: 1.01–2.66 for females).

## Study Limitations

In spite of the encouraging results observed in our study, several potential limitations should be recognized. First, the fact that our study involved individuals of the same ethnicity limits the generalizability of the findings to other regions with different patient populations. Second, as we lacked information about medications and diet control, we did not adjust for these two important factors in the logistic regression models. Third, there is still the possibility of unmeasured confounding factors. Fourth, sequential evaluation of MBCS transition (e.g., semiannually and annually) may reflect the dynamic aspects of clinical status and thus may have provided better information about the risk of RKFD in our participants. In the Whitehall II study, almost 50% people in MHOW group converted to an unhealthy phenotype after 20-year follow-up (40). All-cause mortality and cardiovascular events were also higher in MHOW group than in MHNW group in studies with 10 or more years of follow-up (41). Although our study showed that the risk of RKFD was not statistically significant in the MHOW group even after risk adjustment, the risk of RKFD may have increased gradually with longer follow-up. Fifth, most of the participants were female (70.9%), and the statistically insignificant association between MUNW and RKFD in the male participants may be due to the relatively small sample size in the subgroup of males with MUNW. Sixth, the predictive accuracy of logistic regression models has its own limitations. Finally, this study also could not address the causal effects between MBCS and RKFD.

## Conclusion

In summary, this investigation demonstrated that MBCS was independently associated with RKFD, especially in the older adults. On the basis of our results, we suggest that MUNW and MUOW should be considered as risk factors for RKFD. Timely recognition and further optimization of nephroprotection measures for these subjects may help to slow the decline in renal function.

## Data Availability Statement

The raw data supporting the conclusions of this article will be made available by the authors, without undue reservation.

## Ethics Statement

The studies involving human participants were reviewed and approved by the Institutional Review Board of Chang Gung Memorial Hospital (Approval No. 201800277B0C601). The patients/participants provided their written informed consent to participate in this study.

## Author Contributions

K-YL, C-CL, I-WW, C-YS, H-JH, and H-CP contributed to the conception, design and interpretation of data, provided patient information and participated in the design and coordination, and helped draft the manuscript. S-CC, P-HW, K-YL, C-CK, Y-HS, and H-CP contributed to collecting data, manuscript drafting, provided intellectual content of the work, and were involved in editing and revising the manuscript. All authors discussed, contributed to, and approved the final manuscript version.

## Funding

This study was supported by grants from the Chang Gung Memorial Hospital Research Projects (CMRPG-2G0361, CMRPG-2H0161, CMRPG-2J0261, CMRPG-2K0091, and CLRPG2L0051) and Ministry of Science and Technology (MOST) of the Republic of China (Taiwan) (MOST 106-2314-B-182A-064, MOST 107-2314-B-182A-138, and MOST 108-2314-B-182A-027).

## Conflict of Interest

The authors declare that the research was conducted in the absence of any commercial or financial relationships that could be construed as a potential conflict of interest.

## Publisher's Note

All claims expressed in this article are solely those of the authors and do not necessarily represent those of their affiliated organizations, or those of the publisher, the editors and the reviewers. Any product that may be evaluated in this article, or claim that may be made by its manufacturer, is not guaranteed or endorsed by the publisher.
