# Supplementary material for: Relationships Between Metabolic Body Composition Status and Rapid Kidney Function Decline in a Community-Based Population: A Prospective Observational Study
Source: Front Public Health. 2022 Jun 3;10:895787. doi: 10.3389/fpubh.2022.895787 (PMC9204180; doi:10.3389/fpubh.2022.895787)
Supplement: Supplementary file 1 [file Table_1.pdf]

Supplementary Table 1. Metabolic body composition status classification

	BMI < 24 kg/m^2	BMI ≥ 24 kg/m^2
Metabolically healthy	Metabolically healthy normal weight (MHNW)	Metabolically healthy Overweight (MHOW)
Metabolically unhealthy	Metabolically unhealthy normal weight (MUNW)	Metabolically unhealthy overweight (MUOW)

*Metabolically healthy: HOMA-IR score < 2.5 without metabolic syndrome

*Metabolically unhealthy: HOMA-IR score ≥ 2.5 or with metabolic syndrome
