# Supplementary material for: Relationships Between Metabolic Body Composition Status and Rapid Kidney Function Decline in a Community-Based Population: A Prospective Observational Study
Source: Front Public Health. 2022 Jun 3;10:895787. doi: 10.3389/fpubh.2022.895787 (PMC9204180; doi:10.3389/fpubh.2022.895787)
Supplement: Supplementary file 2 [file Table_2.pdf]

Supplementary Table 2. Social psychology variables of the study population

	Total (n=731)	RKFD (n=125)	No RKFD (n=606)	*p* value
Education level				**0.005**
None, n (%)	31 (4.2%)	12 (9.6%)	19 (3.1%)	
Elementary school, n (%)	172 (23.5%)	34 (27.2%)	138 (22.8%)	
Junior high school, n (%)	118 (16.1%)	24 (19.2%)	94 (15.5%)	
Senior high school, n (%)	246 (33.7%)	36 (28.8%)	210 (34.7%)	
College or university, n (%)	138 (18.9%)	15 (12.0%)	123 (20.3%)	
Graduate school, n (%)	19 (2.6%)	3 (2.4%)	16 (2.6%)	
Substance habits				
Smoking, n (%)	130 (17.7%)	15 (12.0%)	115 (19.0%)	NS (0.072)
Betel nut, n (%)	27 (3.7%)	5 (4.0%)	22 (3.6%)	NS (0.795)
Alcohol, n (%)	178 (24.3%)	22 (17.6%)	156 (25.7%)	**0.036**
Dietary habits				**0.017**
Meat diet, n (%)	641 (87.6%)	101 (80.8%)	540 (89.1%)	
Vegetarian food, n (%)	81 (11.1%)	22 (17.6%)	59 (9.7%)	
Depressive mood, n (%)	95 (13.0%)	27 (21.6%)	68 (11.2%)	**0.003**

Abbreviations: MHNW, metabolically healthy normal-weight; MHOW, metabolically healthy overweight; MUNW, metabolically unhealthy normal-weight; MUOW, metabolically unhealthy overweight
