# Supplementary material for: Relationships Between Metabolic Body Composition Status and Rapid Kidney Function Decline in a Community-Based Population: A Prospective Observational Study
Source: Front Public Health. 2022 Jun 3;10:895787. doi: 10.3389/fpubh.2022.895787 (PMC9204180; doi:10.3389/fpubh.2022.895787)
Supplement: Supplementary file 3 [file Table_3.pdf]

Supplementary Table 3. Combined effects of sex, age, and MBCS on RKFD

	Number	RKFD rate	Unadjusted model		Adjusted model	
			HR (95% CI)	p value	HR (95% CI)	p value
**Total**						
MHNW	322	41 (12.7%)	1 (reference)	1 (reference)	1 (reference)[a]	1 (reference) [a]
MHOW	173	24 (13.9%)	1.09 (0.66-1.80)	0.752	1.08 (0.63-1.85)	0.788
MUNW	56	16 (28.6%)	**2.36 (1.33-4.21)**	**0.004**	**2.49 (1.35-4.60)**	**0.004**
MUOW	180	44 (24.4%)	**2.03 (1.32-3.10)**	**0.001**	**1.79 (1.12-2.86)**	**0.016**

a. Adjusted for education level, alcohol consumption, dietary habits, and depressive mood.
